# Fibrinogen-to-albumin ratio and risk of thrombotic diseases incidence

**DOI:** 10.1186/s12959-026-00867-4

**Published:** 2026-04-28

**Authors:** Bo Zhu, Xinxin Feng, Bolin Li, Rong Fan, Hui Liu, Xinping Kang, Shuyi Deng, Lele Cheng, Zuyi Yuan, Yue Wu, Tao Zheng

**Affiliations:** 1 Department of Cardiology, The Affiliated Hospital of Southwest Medical University, Luzhou, Sichuan 646000 China; 2 Department of Cardiology, The First Affiliated Hospital of Xi’an Jiaotong University, 277 West Yanta Road, Xi’an, Shaanxi 710061 China; 3 Department of Cardiology, Xi’an Central Hospital, Xi’an, Shaanxi 710061 China; 4 Department of Cardiology, The Third People’s Hospital of Chengdu, Affiliated Hospital of Southwest Jiaotong University, Chengdu, Sichuan 610014 China; 5 Biobank, The First Affiliated Hospital of Xi’an Jiaotong University, 277 West Yanta Road, Xi’an, Shaanxi 710061 China; 6 Fifth Outpatient Department, Xijing 986 Hospital, Xi’an, Shaanxi 710054 China; 7 Department of Cardiology, The First Affiliated Hospital of Xi’an Jiaotong University, No. 76, Yanta West Road, Xi’an, Shaanxi 710061 China

**Keywords:** Fibrinogen-to-albumin ratio, Arterial and venous thrombosis, Acute myocardial infarction, Stroke, Pulmonary embolism, Deep venous thrombosis

## Abstract

**Background:**

The fibrinogen-to-albumin ratio (FAR) is linked to cardiovascular diseases, but its association with thrombotic diseases in the general population remains unclear.

**Objective:**

To investigate the relationship between FAR and thrombotic diseases and assess their dose-response association.

**Methods:**

This prospective cohort study included 18,208 participants (mean age 53.2 years, 41.9% female) from the Xifei community cohort (2011–2021). Participants were categorized into FAR quintiles (Q1–Q5). Restricted cubic spline and Cox regression models were used to assess the associations between FAR and thrombotic risk.

**Results:**

Over follow-up of 8 years, 1,373 thrombotic events occurred. Compared to the group of lowest FAR, the group of highest FAR showed significantly increased risks of combined arterial and venous thrombosis (HR = 1.36, 95% CI 1.08–1.70), arterial thrombosis (HR = 1.35, 1.07–1.70), and stroke (HR = 1.36, 1.06–1.76). Risks for acute myocardial infarction, venous thromboembolism, deep venous thrombosis, and pulmonary embolism were not statistically significant. A nonlinear dose-response relationship between FAR and thrombotic risk was observed.

**Conclusions and relevance:**

Higher baseline FAR was associated with an elevated risk of arterial and venous thrombosis, arterial thrombosis, and stroke, whereas associations with acute myocardial infarction and venous outcomes (VTE, DVT, and PE) were not statistically significant. These findings suggest that FAR may be a useful biomarker for thrombotic risk assessment in the general population.

**Supplementary Information:**

The online version contains supplementary material available at 10.1186/s12959-026-00867-4.

## Research insights

What is currently known about this topic?

What is the key research question?

What is new?

How might this study influence clinical practice?

Fibrinogen-to-Albumin Ratio (FAR) has been demonstrated in numerous studies to be associated with various cardiovascular diseases. However, there remains a significant gap in our understanding of the relationship between FAR and the incidence of thrombotic disorders in the general population, particularly in the absence of large-scale prospective clinical research with long-term follow-up.

Is baseline FAR associated with the risk of incident thrombotic diseases in a community-based general population?

In this prospective community-based cohort study, higher baseline FAR was associated with a modestly increased risk of arterial thrombotic outcomes, particularly arterial thrombosis and stroke. Associations with acute myocardial infarction and venous thromboembolic events were not consistently significant after full adjustment and should be interpreted cautiously.

These findings suggest that FAR, as a composite biomarker derived from routine laboratory parameters, may help identify individuals at higher risk of arterial thrombotic events in the general population. Further studies are needed to confirm its potential role in risk stratification and clinical decision-making.

**Supplementary Information:**

The online version contains supplementary material available at 10.1186/s12959-026-00867-4.

## Introduction

Thrombosis, which refers to localized blood clotting, can occur in either the arterial or venous circulation and has a significant medical impact. Acute arterial thrombosis is the primary cause of most cases of myocardial infarction (MI) and of about 80% of strokes. Deep venous thrombosis (DVT) and pulmonary embolism (PE) constitute venous thromboembolism (VTE), which is the third leading cause of cardiovascular-related death [1]. Because many thrombotic events are preventable, maintaining a delicate balance between bleeding and coagulation, and taking appropriate preventive measures, is crucial [2]. In the past, multiple biomarkers and subsequent models have been developed to assess the risk of arterial or venous thrombosis in patients [3–7]. However, reliable and simple biomarkers for thrombotic events in the general population are still lacking. In the context of COVID-19 and oncological diseases, the incidence of thrombotic disorders, including both arterial and venous thrombosis, increases, suggesting that COVID-19 and oncological conditions share many common mechanisms underlying the development of both arterial and venous thrombotic diseases [8–12].

Fibrinogen (FIB), participates in platelet aggregation and activation [13, 14], is also an indicator for a proinflammatory state [15] and a high-risk marker for developing vascular inflammatory diseases, such as hypertension and atherosclerosis [16, 17]. Many observational studies have found that plasma FIB levels are independently associated with cardiovascular diseases [16–19] including MI [20], [21], stroke [20, 22] and VTE [23]. Furthermore, thrombotic risk is also increased in settings such as COVID-19 infection, highlighting the importance of inflammation-related pathways in thrombogenesis [11]. Albumin (ALB), while frequently used as a marker of nutritional status [24, 25] or hepatic function [26], serves as a well-recognized indicator of inflammatory status [27], and predicts cardiovascular mortality [28].

More researchers have begun to focus on the role of FIB, ALB and fibrinogen-to-albumin ratio (FAR), which combines the aforementioned two indices, in patients with specific thrombotic diseases in recent years [29–32]. We speculate that FAR may provide additional information beyond FIB or ALB alone for thrombotic risk assessment from an inflammatory and nutritional perspective. If validated, this may support population-level risk stratification and prevention.

However, previous studies have mainly focused on patients with specific diseases, and the majority of these studies had small sample sizes. Currently, there is a lack of large sample studies on the correlation between FAR with overall incidence of both arterial and venous thrombotic events combined in the general population. Therefore, our large-scale prospective cohort study aims to address this gap by examining the relationship between FAR and the risk of overall thrombotic diseases as a whole in a general community population with 8-year follow-up.

## Methods

### Study population

This study is a large-scale prospective cohort study of general population (aged 18–100 years) living in Yanliang community in Shaanxi Province in China, named Xifei prospective cohort study. In this cohort, a total of 18 208 participants were included, all of whom had complete data on serum FIB and ALB concentration, creatinine (Crea), blood urea nitrogen (BUN), total cholesterol (TC), triglycerides (TG), high-density lipoprotein (HDL) and low-density lipoprotein (LDL). We excluded individuals who: (A) lacked FIB or ALB values, (B) had a history of thrombotic disorders, autoimmune diseases, fractures, or malignancy (eFigure 1). Information about participants was collected through questionnaires, physical measurements, and biological samples during the follow up period. We obtained oral informed consent by telephone from all participants and written informed consent from clinicians. After baseline examinations, follow-up examinations took place approximately every 2 years during a total follow-up of 8 years. This study adhered to the principles of the Declaration of Helsinki and received approval from the Ethics Committee of the First Affiliated Hospital of Xi’an Jiaotong University (Ethical approval number: XJTU1AFCRC2020SJ-018).

### Measurement of FAR

The FAR was defined as the ratio of the fibrinogen level to the albumin level. We combined FIB and ALB into a composite marker. The serum fibrinogen levels were measured using the coagulation method with the Werfen fibrinogen kit (Instrumentation Laboratory, USA), and the albumin concentrations were determined using colorimetry (Roche Diagnostics GmbH, Germany). Both measurements were conducted within 24 h of collecting peripheral blood samples on an empty stomach in the morning. Extensive quality control was performed, and all biochemical variables were assayed using an autoanalyzer at the central laboratory of the YanLiang Community Hospital.

### Outcomes

In this study, the main outcomes were arterial and venous thrombosis, composed of acute myocardial infarction (AMI), stroke, PE, and DVT. Among them, AMI and stroke were classified as arterial thrombosis, PE and DVT were classified as VTE. We used the *International Statistical Classification of Diseases*,* Tenth Revision* (ICD 10), to identify above thrombotic diseases. The participants were enrolled in 2011–2013 (*n *=18776) and followed-up in 2013–2015 (visit 1, *n* = 18278), 2015–2017 (visit 2, *n* = 16871), 2017–2019 (visit 3, *n* = 17954), and 2019–2021 (visit 4, *n* = 16308). Retention rates have been high throughout the 8 years of follow-up (97.35%, 89.85%, 95.62%, 86.86%, respectively).

### Covariates

In this study, covariates included demographic characteristics, physiological parameters, laboratory tests, and clinical information. The demographic characteristics, which were collected through a self-report questionnaire, included age at baseline and sex. Physiological parameters included body mass index (BMI; calculated as weight in kilograms divided by height in meters square) and blood pressure (BP; measured to the nearest 1 mmHg with mercury sphygmomanometers using standard recommended procedures). The BMI was used to divide participants into underweight (BMI, < 18.5), normal weight (BMI, 18.5–24.9), overweight (BMI, 25.0-29.9), and obese (BMI, ≥ 30.0) groups. Laboratory tests including Crea, BUN, TC, TG, HDL, and LDL levels (measured using an autoanalyzer within 24 h of peripheral blood collected on an empty stomach in the morning, with extensive quality control performed by the hospital of YanLiang community). Clinical information, including the presence of hypertension and diabetes mellitus, was obtained from self-reports by participants or proxies. Hypertension was defined as if any of the following conditions was true: (i) BP ≥ 140/90 mmHg; (ii) use of antihypertensive drugs; (iii) self-reported hypertension history [33, 34]. Diabetes mellitus (DM) was defined as if any of the following conditions was true: (i) fasting blood glucose (FBG) ≥ 7.0 mmol/L; (ii) use of hypoglycaemic drugs; (iii) self‐reported DM history [34–38].

### Statistical analysis

First, we used the frequency and percentage for categorical variables and the mean (SD) or median (IQR) for continuous variables. The analysis of variance (ANOVA) was employed to assess differences in parameters among groups for normally distributed variables. For non-normally distributed continuous variables, the Kruskal-Wallis test was applied, while the chi-square test was utilized for categorical variables. And we used multiple imputation by chained equations to address the missing data. In our dataset, approximately 11% of the covariate data were missing, and we addressed this using Multiple Imputation (MI) under the assumption that data were missing at random (MAR). The imputations were performed with the mice package in R, generating five imputed datasets that included all variables in the analysis model. Continuous variables were imputed using Predictive Mean Matching (PMM), and categorical variables were imputed using polytomous logistic regression. All reported results in the manuscript are based on these pooled analyses, ensuring robustness and minimizing bias due to missingness. Second, we conducted a Kaplan-Meier survival analysis to plot the associations of different FAR groups with thrombotic diseases. Third, we also conducted restricted cubic spline regressions on FAR as the continuous variable to estimate possible nonlinearity between FAR and the risk of thrombotic diseases. Fourth, we categorized the FAR levels into 5 quintiles (Qs; Q1, 0.054–0.063; Q2, > 0.063–0.067; Q3, > 0.067–0.071; Q4, > 0.071–0.075; and Q5, > 0.075–0.089) and then used Cox proportional hazards regression models to estimate the hazard ratios (HRs) and 95% confidence intervals (CIs) of FAR levels associated with thrombotic diseases using 3 models: model 1 was the crude model, model 2 was adjusted for age and sex, and model 3 was adjusted for age, sex, BMI, BP, Crea, BUN, TC, TG, HDL, LDL, hypertension, and DM. Fifth, subgroups were created according to age group (< 45, 45–64, and ≥ 65 years), sex, BMI category (< 18.5, 18.5–24.9, 25.0-29.9, ≥ 30.0), and hypertension. And their interaction effect was estimated by the Wald test. All results were considered significant at *P* < 0.05 (2-tailed). All analyses were conducted using R statistical software, version 4.2.1 (R Project for Statistical Computing).

## Results

### Characteristics of the study population

Table 1 shows the characteristics of the study population in YanLiang Community. The baseline demographic characteristics, physiological parameters, and health history of the study population are presented in detail. At baseline, 18,208 participants (41.9% female and 58.1% male) in this cohort were categorized into 5 groups based on quintiles, the mean (SD) age was 53.22 (13.60) years. The mean (SD) FAR levels were 0.069 (0.007). In addition, 1,373 arterial and venous thrombosis, including 1298 arterial thrombosis, 143 VTE, 202 AMI, 1152 stroke, 42 PE, and 119 DVT over a total follow-up of 8 years were documented. As indicated in Table 1, population with higher FAR values were older, with more women and higher BMI, and had a higher prevalence of DM, a higher levels of Crea, BUN, TC, TG, HDL, LDL (all *P* < 0.001) as well than participants with lower FAR values. SBP and DBP did not differ among the five groups.

**Table 1:** Baseline characteristics of participants according to FAR values

Variables	Q1(0.054 ≤ FAR < 0.063, *n* = 3642)	Q2(0.063 ≤ FAR < 0.067, *n* = 3642)	Q3(0.067 ≤ FAR < 0.071, *n* = 3642)	Q4(0.071 ≤ FAR < 0.075, *n* = 3641)	Q5(0.075 ≤ FAR < 0.089, *n* = 3642)	*P* Value
Age, years	45.92 (10.15)	49.59 (12.63)	53.42 (13.99)	57.00 (15.18)	60.17 (16.05)	< 0.001
Female, %	1273 (35.0)	1266 (34.8)	1472 (40.4)	1639 (45.0)	1972 (54.1)	< 0.001
BMI, kg/m^2^	23.29 (3.04)	23.50 (3.04)	23.64 (3.04)	23.65 (3.00)	23.65 (3.07)	< 0.001
SBP, mmHg	118.53 (13.24)	120.86 (14.58)	139.68 (10.00)	148.75 (11.75)	143.30 (91.77)	0.377
DBP, mmHg	75.60 (8.89)	95.76 (11.62)	77.01 (11.77)	77.54 (17.68)	77.09 (8.83)	0.419
Crea, umol/L	66.86 (14.10)	69.37 (22.44)	69.22 (16.30)	69.00 (15.15)	69.23 (23.08)	< 0.001
BUN, mmol/L	4.74 (1.13)	4.82 (1.13)	4.83 (1.12)	4.86 (1.11)	4.87 (1.17)	< 0.001
TC, mmol/L	4.48 (0.84)	4.60 (0.85)	4.73 (0.87)	4.91 (0.93)	5.03 (0.97)	< 0.001
TG, mmol/L	1.55 (1.10)	1.64 (1.16)	1.71 (1.20)	1.70 (1.12)	1.76 (1.33)	< 0.001
HDL, mmol/L	1.41 (0.30)	1.41 (0.30)	1.42 (0.31)	1.43 (0.31)	1.44 (0.32)	< 0.001
LDL, mmol/L	2.13 (0.59)	2.16 (0.58)	2.20 (0.58)	2.26 (0.59)	2.27 (0.60)	< 0.001
DM, %	33 (0.9)	32 (0.9)	51 (1.4)	83 (2.3)	111 (3.0)	< 0.001
AV, %	106 (2.9)	201 (5.5)	243 (6.7)	361 (9.9)	462 (12.7)	< 0.001
AT, %	100 (2.7)	190 (5.2)	232 (6.4)	343 (9.4)	433 (11.9)	< 0.001
AMI, %	21 (0.6)	32 (0.9)	22 (0.6)	58 (1.6)	69 (1.9)	< 0.001
Stroke, %	82 (2.3)	164 (4.5)	218 (6.0)	304 (8.3)	384 (10.5)	< 0.001
VTE, %	12 (0.3)	15 (0.4)	21 (0.6)	39 (1.1)	56 (1.5)	< 0.001
DVT, %	12 (0.3)	12 (0.3)	16 (0.4)	32 (0.9)	47 (1.3)	< 0.001
PE, %	1 (0.0)	3 (0.1)	9 (0.2)	10 (0.3)	19 (0.5)	< 0.001

Continuous variables were reported as mean (SD), and categorical variables were reported as frequencies and percentages

FAR: fibrinogen-to‐albumin ratio; BMI: body mass index; SBP: systolic blood pressure; DBP: diastolic blood pressure; Crea: creatinine; BUN: blood urea nitrogen; TC: total cholesterol; TG: triglycerides; HDL: high-density lipoprotein cholesterol; LDL: low-density lipoprotein cholesterol; DM: diabetes mellitus; AV: arterial and venous thrombosis; AT: arterial thrombosis; AMI: acute myocardial infarction; VTE: venous thromboembolism; DVT: deep venous thrombosis; PE: pulmonary embolism. Quartiles of FAR, Q1: 0.054 ≤ FAR < 0.063, Q2: 0.063 ≤ FAR < 0.067, Q3: 0.067 ≤ FAR < 0.071, Q4: 0.071 ≤ FAR < 0.075, and Q5: 0.075 ≤ FAR < 0.089

We conducted a comparison of arterial and venous thrombosis among the 5 groups divided by different FAR levels (eFigure 2). As shown in the figure, with increased FAR levels, the risk of arterial and venous thrombosis increases. Compared with participants in the lowest quintile of FAR, those in the highest quintile of FAR demonstrated the highest incidence of arterial and venous thrombosis, and even when stratified by different numbers of thrombotic events, those in higher quintiles of FAR consistently exhibited higher incidence of arterial and venous thrombosis.

### Association between FAR and the risk of thrombotic diseases

**Fig. 1:**
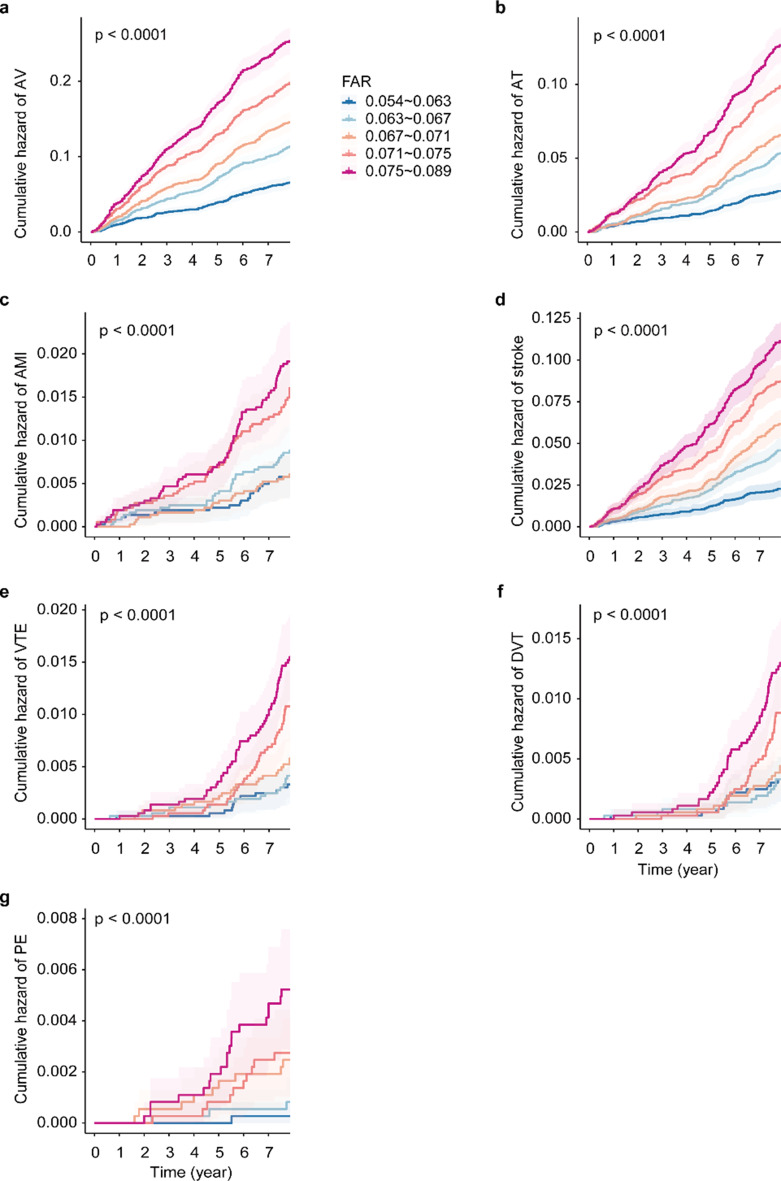
Cumulative risk of thrombotic diseases stratified by FAR values. (**a**) Cumulative risk of arterial and venous thrombosis stratified by FAR values. (b) Cumulative risk of arterial thrombosis stratified by FAR values. (**c**) Cumulative risk of acute myocardial infarction stratified by FAR values. (d) Cumulative risk of stroke stratified by FAR values. (**e**) Cumulative risk of venous thromboembolism stratified by FAR values. (**f**) Cumulative risk of deep venous thrombosis stratified by FAR values. (**g**) Cumulative risk of pulmonary embolism stratified by FAR values

Kaplan-Meier survival plots indicated that participants in the higher FAR quintiles had significantly greater cumulative risk of arterial and venous thrombosis, as well as stroke, AMI, VTE, DVT, and PE than those in the lowest quintiles. For example, compared with participants in the lowest quintile of FAR, those in the highest quintile of FAR show the highest cumulative incidence of arterial and venous thrombosis, arterial thrombosis, VTE, stroke, AMI, PE and DVT after a follow-up of 8 years, respectively. Furthermore, the longer the follow-up, the greater the risk of thrombotic diseases (Fig. 1).

**Fig. 2:**
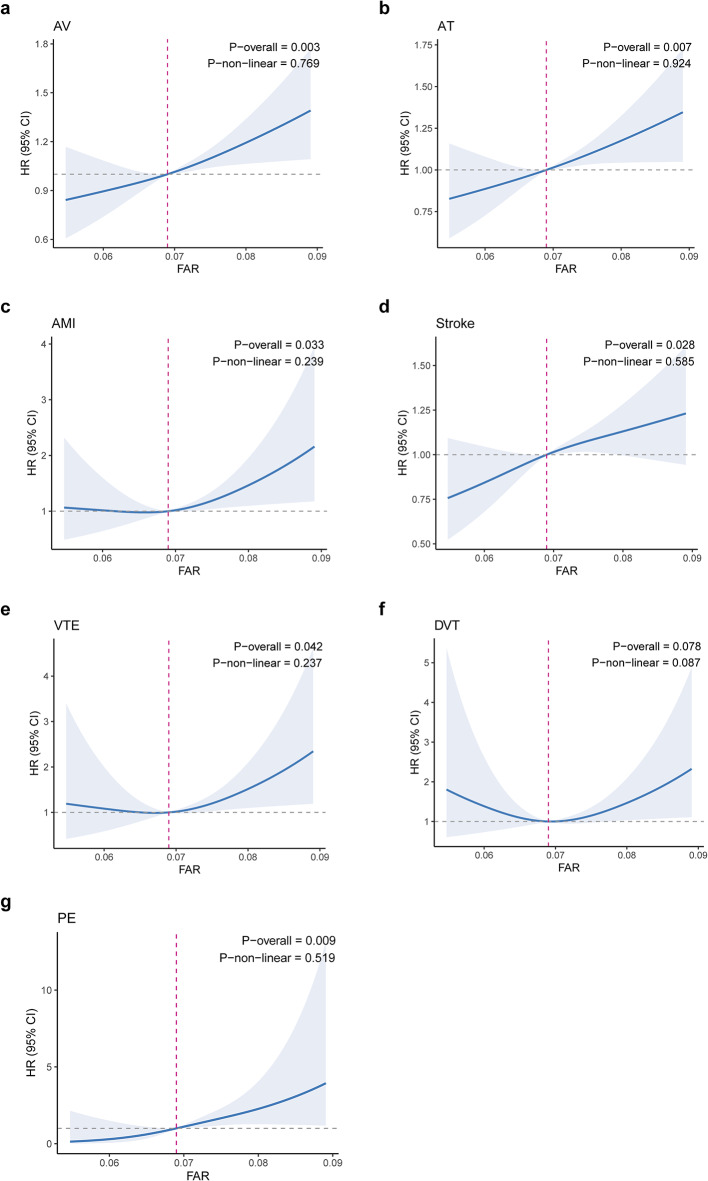
Associations between FAR values and thrombotic diseases in the participants. HR: hazard ratio; The data were plotted based on results from adjusted Cox models or Fine and Gray subdistribution hazard models for FAR values using penalized cubic splines. The shading indicates the 95% CIs. The corresponding reference values vary per graph and are used specifically for each FAR and event combination. (The hazard ratio is represented by a bold line; the 95% confidence interval is represented by the shaded area)

We used cubic spline regression to evaluate the dose-response association between FAR and risk of arterial and venous thrombosis, modeling FAR as a continuous variable. We found a nonlinear, positive association between FAR and risk of all the thrombotic diseases, especially, arterial and venous thrombosis, arterial thrombosis stroke and pulmonary embolism in this cohort (Fig. 2).

**Fig. 3:**
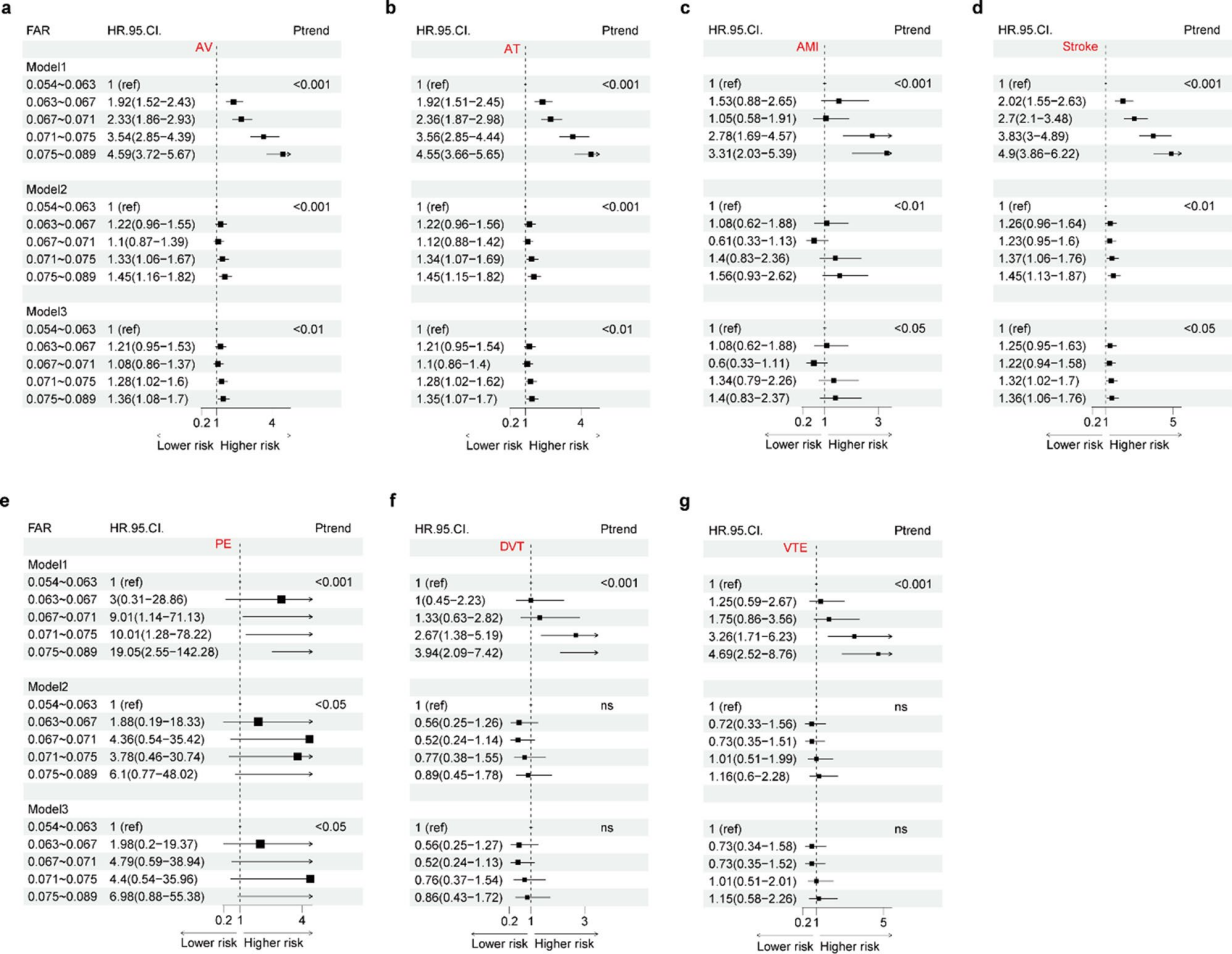
Association of FAR values with thrombotic diseases in population. (**a**) Association of FAR values with arterial and venous thrombosis. (**b**) Association of FAR values with arterial thrombosis. (**c**) Association of FAR values with acute myocardial infarction. (**d**) Association of FAR values with stroke. (**e**) Association of FAR values with venous thromboembolism. (**f**) Association of FAR values with deep venous thrombosis. (**g**) Association of FAR values with pulmonary embolism. HR, hazard ratio; Model 1: unadjusted. Model 2: adjusted for age and sex. Model 3: adjusted for the same risk factors as adjusted model 2 plus BMI, Crea, BUN, TC, TG, HDL, LDL, hypertension and DM

Cox proportional hazards regression analyses show that higher FAR levels were significantly associated with higher risk of all the thrombotic diseases. Higher FAR levels were positively associated with arterial and venous thrombosis, as well as arterial thrombosis, stroke, PE, AMI, VTE, and DVT. For example, compared with participants in the lowest quintile of FAR, those in the highest quintile of FAR experienced the highest risk of arterial and venous thrombosis (HR, 4.59 [95% CI, 3.72–5.67]), arterial thrombosis (HR, 4.55 [95% CI, 3.66–5.65]), VTE (HR, 4.69 [95% CI, 2.52–8.76]), stroke (HR, 4.9 [95% CI, 3.86–6.22]), AMI (HR, 3.31 [95% CI, 2.03–5.39]), PE (HR, 19.05 [95% CI, 2.55-142.28]) and DVT (HR, 3.94 [95% CI, 2.09–7.42]), respectively. And for arterial and venous thrombosis, arterial thrombosis and stroke, the associations remained significant even after adjusting for age, sex, hypertension, DM, BMI, Crea, BUN, TC, TG, HDL and LDL, but for AMI, VTE, DVT, and PE, the results were not similar. For example, compared with participants in the lowest quintile of FAR, those in the highest quintile of FAR were associated with 1.36-fold risk of arterial and venous thrombosis, 1.35-fold risk of arterial thrombosis and 1.36-fold risk of stroke (Fig. 3).

In addition, we conducted stratified analyses of this cohort by age group, sex, BMI category, and hypertension. Elevated FAR levels were associated with a higher risk of arterial and venous thrombosis across strata. Similar patterns were observed for arterial thrombosis and stroke, and were directionally similar for PE, although estimates were imprecise due to the small number of events. Overall, these results suggest potential heterogeneity in the association between FAR and thrombotic outcomes across subgroups defined by age, sex, hypertension status, and BMI categories. For example, participants with hypertension, older age, higher BMI, and females appeared to have higher risks compared with their corresponding reference groups (eFigure 3). These subgroup and interaction findings are exploratory and should be interpreted cautiously due to multiple testing.

## Discussion

To our knowledge, this cohort study is the largest study, based on a long-term follow-up cohort of natural community-based populations, in which the association of FAR with various thrombotic diseases was examined.

Using data from YanLiang community, involving 18,208 participants at baseline and 1,373 thrombotic events over 8 years of follow-up, we found that higher FAR levels were associated with the risk of arterial outcomes (arterial thrombosis and stroke); associations with venous outcomes (VTE/DVT/PE) were less consistent after full adjustment and should be interpreted cautiously given the limited number of events. We also observed stronger associations of increased FAR levels with the risk of arterial and venous thrombosis, arterial thrombosis, and stroke, for example, compared with participants in the lowest quintile of FAR, those in the highest quintile of FAR were associated with 1.36-fold risk of arterial and venous thrombosis, 1.35-fold risk of arterial thrombosis and 1.36-fold risk of stroke, whereas the associations with AMI and venous outcomes (VTE/DVT/PE) were not statistically significant after full adjustment. Furthermore, significant interaction effects were observed between hypertension and FAR levels on arterial and venous thrombosis, arterial thrombosis, and stroke. Participants with hypertension exhibited a greater risk compared to those without hypertension, suggesting that the association between FAR and thrombotic outcomes may differ by hypertension status. These findings deepened our understanding of the risk factors associated with systemic thrombotic diseases in the community population, providing valuable references for the management and prevention of such disorders.

Numerous studies have assessed the role of FAR in predicting the prognosis of some specific thrombotic diseases in various disease-specific patients. Less attention, however, has been paid to the occurrence of thrombotic diseases in the general population, which can be avoided by effective early intervention. Thus, estimating reliable circulating biomarkers has significant meaning to the prevention and management of thrombotic diseases.

Previous studies have examined the association of FAR with some specific thrombotic diseases in various disease-specific populations, such as patients with AMI [39, 40], atrial fibrillation [41], diabetic kidney disease [42], heart failure [43, 44], stroke [45, 46], and cancer [47]. Published studies have mainly focused on various disease-specific populations, the majority of which had small sample sizes, and indicated significant associations of FAR with the prognosis of thrombotic events. Compared with the results of previous studies, we examined the association of FAR levels with the risk of thrombotic diseases in a large-scale, diverse, community-based cohort study of a general population in China. Our study is the first systematic exploration of the correlation between FAR and the occurrence of thrombotic diseases and provides strong epidemiologic evidence that elevated FAR levels are associated with an increased risk of thrombotic diseases in the general population. Furthermore, different from past research, we observed that the association between FAR and thrombotic diseases varied with the conditions of hypertension, age, BMI, and sex. High FAR was associated with a higher risk of thrombotic diseases in people with a history of hypertension, older age, higher BMI, and female gender.

The biological mechanisms underlying the association between higher FAR and thrombotic diseases likely involve a complex interplay between inflammation, coagulation, and platelet activation. FIB, a soluble glycoprotein mainly synthesized in the liver, serves as both an acute-phase reactant and a key mediator of thrombogenesis. Elevated FIB promotes platelet aggregation and activation, enhances smooth muscle cell proliferation, and upregulates the expression of cell adhesion molecules and pro-inflammatory cytokines [15, 18]. These effects contribute not only to the formation of a hypercoagulable state but also to the initiation and progression of atherosclerotic plaques [19, 20, 23, 48–50]. Moreover, FIB interacts directly with activated platelets, facilitating thrombus growth and stability, thereby linking inflammatory responses to thrombotic events. ALB, the most abundant plasma protein, exerts anti-inflammatory and antioxidant effects, and can inhibit platelet aggregation, thus reducing plasma viscosity [51]. Low ALB levels may trigger compensatory mechanisms, including increased synthesis of procoagulant factors and lipoproteins, which lead to hypercoagulability and endothelial dysfunction [52]. Hypoalbuminemia also exacerbates oxidative stress and inflammation, further amplifying platelet reactivity and promoting thrombus formation [53]. Taken together, a high FAR, reflecting both elevated FIB and low ALB, represents an integrated marker of a prothrombotic milieu. It embodies heightened inflammatory activity, enhanced platelet activation, and coagulation imbalance, which together increase the risk of arterial and venous thrombotic events. This integrated pathophysiological framework provides mechanistic insights linking inflammation, coagulation, and platelet-related pathways to thrombotic disease development [54]. Our findings demonstrate an association between higher FAR levels and increased risk of arterial thrombotic events in a large community-based cohort. Given the prospective design, these results suggest a potential causal relationship, but residual confounding cannot be ruled out. FAR may represent a candidate biomarker for identifying individuals at higher thrombotic risk; however, its clinical utility requires further validation in independent cohorts and interventional studies before routine implementation.

### Strengths and limitations

Our study boasts several key strengths. Firstly, being a community-based cohort study focused on Chinese population, our findings should be cautiously extrapolated to other ethnic groups. Additional studies encompassing diverse ethnic populations are required to validate our observations. Secondly, all administered questionnaires were self-report measures, which are liable to self-report bias. In addition, venous outcomes (e.g., PE) were relatively rare, which may have resulted in imprecise estimates. Thirdly, although we controlled for many potential confounders, including demographic factors, physiological parameters, and health history, data on concomitant pharmacological treatments, such as antiplatelet agents, anticoagulants, and statins, were not consistently available and could not be included in our multivariable models. Given the well-established effects of these therapies on thrombotic risk and inflammatory/coagulation pathways, residual confounding from medication use may have influenced the observed associations between FAR and thrombotic outcomes. Therefore, the associations reported in this study should be interpreted as observational and associative, highlighting the need for further research with comprehensive medication data to clarify the potential influence of pharmacological confounders.

## Conclusion

This cohort study found that the FAR was associated with an increased risk of arterial and venous thrombosis, arterial thrombosis and stroke, whereas associations with AMI, VTE, DVT, and PE were not statistically significant in the general population. These findings deepened our understanding of the risk factors associated with systemic thrombotic diseases in the community population, providing valuable references for the management and prevention of such disorders. Future studies are needed to validate these findings and to evaluate the clinical utility of FAR for risk stratification.

## Supplementary Information

Below is the link to the electronic supplementary material.

Supplementary Material 1

## Data Availability

The dataset used in this study is available when the publication is online from the corresponding authors (email: zhengtao900305@xjtu.edu.cn) on reasonable request without any additional restrictions.

## Abbreviations

FAR: Fibrinogen-to-albumin ratio
MI: Myocardial infarction
VTE: Venous thromboembolism
DVT: Deep venous thrombosis
PE: Pulmonary embolism
FIB: Fibrinogen
ALB: Albumin
Crea: Creatinine
BUN: Blood urea nitrogen
TC: Total cholesterol
TG: Triglycerides
HDL: High-density lipoprotein
LDL: Low-density lipoprotein
PT: Prothrombin time
APTT: Activated partial thromboplastin time
AMI: Acute myocardial infarction
ICD 10: International Statistical Classification of Diseases, Tenth Revision
BMI: Body mass index
BP: Blood pressure
FBG: Fasting blood glucose
DM: Diabetes mellitus
ANOVA: Analysis of Variance
HR: Hazard ratio
CIs: Confidence intervals
LAA: Large artery atherosclerosis
CAG: Coronary angiography
ACS: Acute coronary syndrome
GS: Gensini score
STEMI: ST-elevation myocardial infarction
OR: Odds ratio
