# Supplementary material for: Fibrinogen-to-albumin ratio and risk of thrombotic diseases incidence
Source: Thromb J. 2026 Apr 28;24:56. doi: 10.1186/s12959-026-00867-4 (PMC13262508; doi:10.1186/s12959-026-00867-4)

**Supplemental Online Content**

**eMethods**

**eFigure 1. Flowchart of study population.**

**eFigure 2. Histograms show the prevalence of arterial and venous thrombosis in the participants stratified by FAR values and the number of events.**

**eFigure 3. Associations between FAR values and thrombotic diseases in the participants.**

**eFigure 4. Interaction effect between FAR and gender and BMI on the risk of thrombotic diseases.**

This supplemental material has been provided by the authors to give readers additional information about their work.

**eFigure 1. Flowchart of study population.**


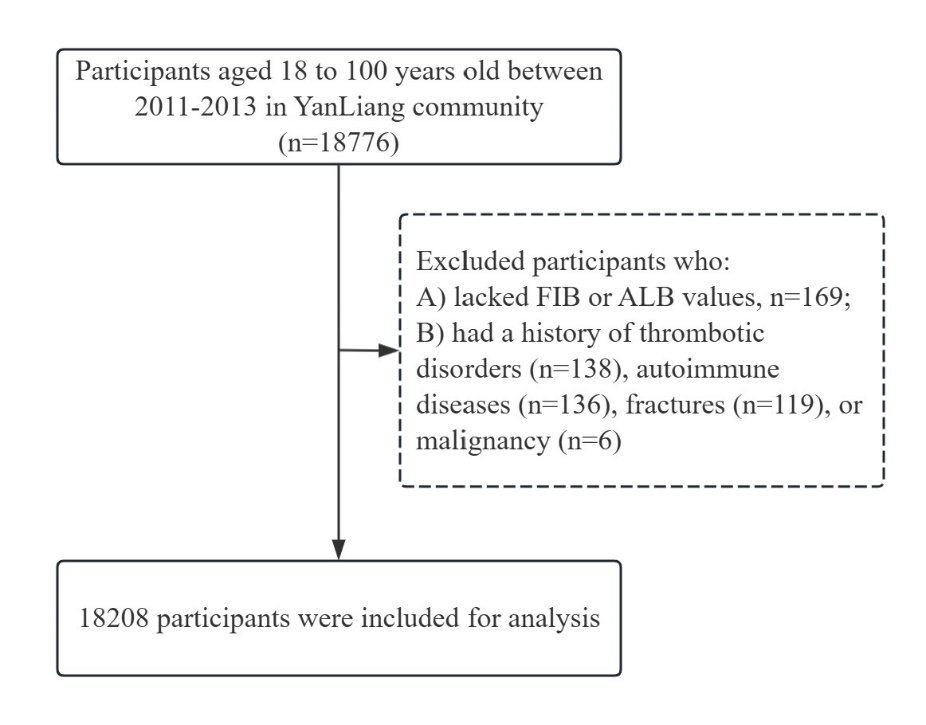


**eFigure 2. Histograms show the prevalence of arterial and venous thrombosis in the participants stratified by FAR values and the number of events.**


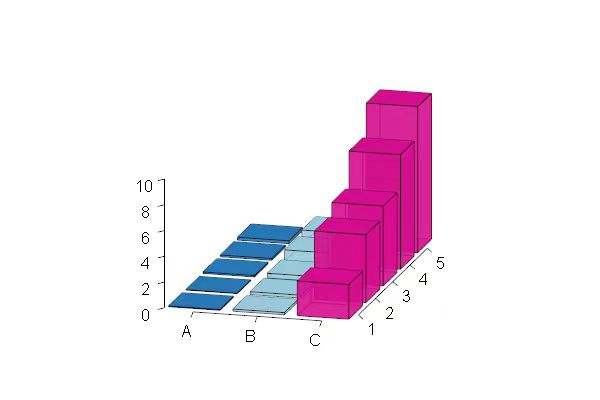


1-5 represent the quartiles of FAR, A-C represent the number of events.

**eFigure 3. Associations between FAR values and thrombotic diseases in the participants.**


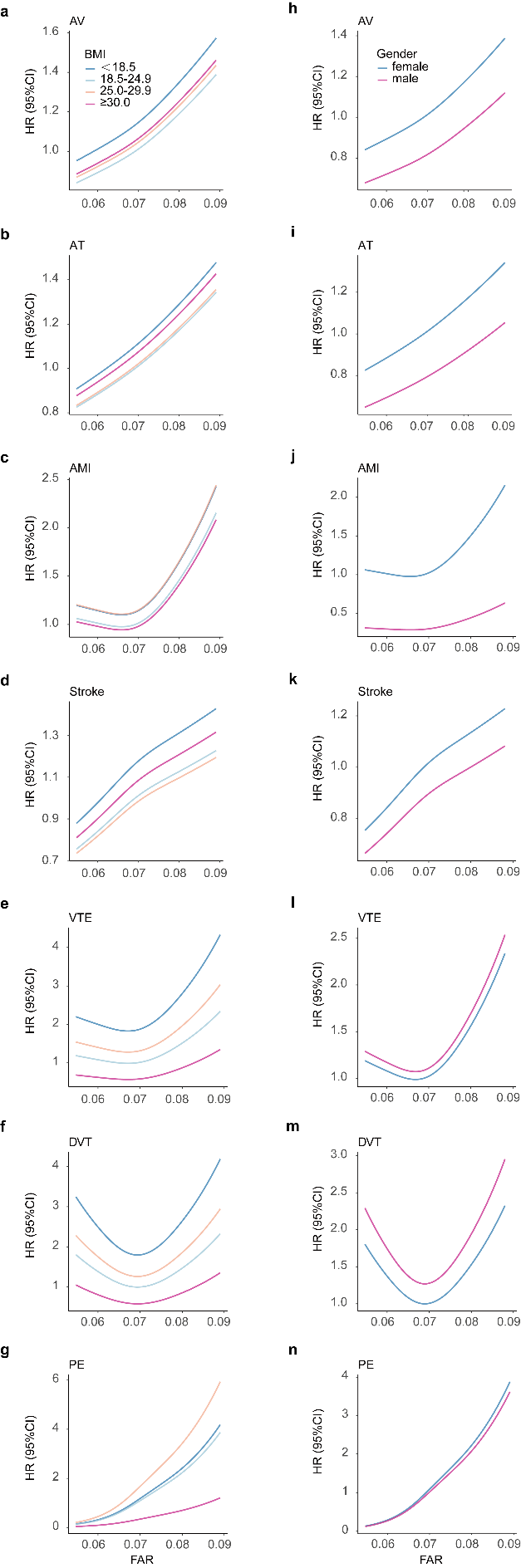


(a-g). Associations between FAR values and thrombotic diseases in the participants stratified by BMI. (h-n). Associations between FAR values and thrombotic diseases in the participants stratified by gender. FAR: fibrinogen‐to‐albumin ratio; AV: arterial and venous thrombosis; AT: arterial thrombosis; AMI: acute myocardial infarction; VTE: venous thromboembolism; DVT: deep venous thrombosis; PE: pulmonary embolism; HR: hazard ratio; CI: confidence interval

**eFigure 4. Interaction effect between FAR and gender and BMI on the risk of thrombotic diseases.**


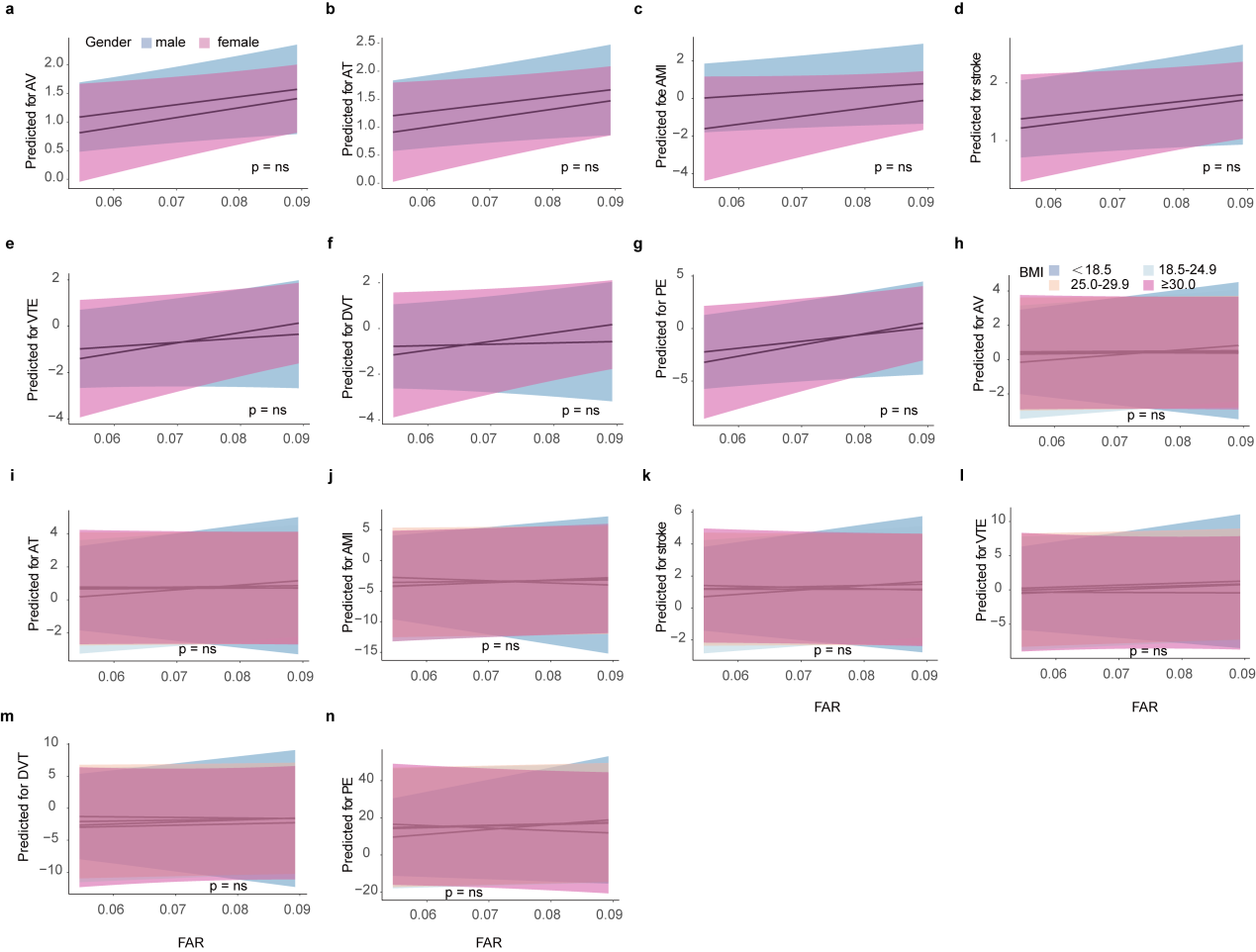

Supplement: Supplementary file 1 — Supplementary Material 1 [file 12959_2026_867_MOESM1_ESM.docx]
